# Report of a patient with a de novo non-recurrent duplication of 17p11.2p12 and Yq11 deletion

**DOI:** 10.1186/s13039-019-0438-0

**Published:** 2019-08-01

**Authors:** Liliana Fernández-Hernández, María José Navarro-Cobos, Miguel Angel Alcántara-Ortigoza, Sandra Elena Ramos-Ángeles, Bertha Molina-Álvarez, Sinhué Díaz-Cuéllar, Bárbara Asch-Daich, Ariadna González-del Angel

**Affiliations:** 1 Laboratorio de Biología Molecular, Departamento de Genética Humana, Instituto Nacional de Pediatría, Ciudad de México, México; 2 Centro de Cirugía Especial de México, Institución de Asistencia Privada, Ciudad de México, México; 3 Centro de Alta Especialidad en Genética Humana, DNA GEN, S.C, Ciudad de México, México; 4 Laboratorio de Citogenética, Departamento de Genética Humana, Instituto Nacional de Pediatría, Ciudad de México, México; 5 Departamento de Genética Humana, Instituto Nacional de Pediatría, Ciudad de México, México; 6 Diagen, Diagnóstico Genético, Ciudad de México, México

**Keywords:** Yq11 deletion, Duplication of 17p11.2p12, Chromosomal microarray analysis, Non-recurrent rearrangements, Concurrent de novo rearrangements

## Abstract

**Background:**

The 17p11.2p12 locus is an unstable region that is predisposed to several known genomic disorders and non-recurrent rearrangements that yield varied and wide-ranging phenotypes. Nearly 1% of male newborns have deletions in the Y chromosome; these events primarily involve the heterochromatic region, but may extend to euchromatic Yq segments containing azoospermia factor regions.

**Case presentation:**

We describe the occurrence of two independent chromosomal rearrangements that originated as de novo events in a single male patient: a 10.8-Mb duplication of 17p11.2p12 and a 14.7-Mb deletion of Yq11. This individual shares some clinical characteristics with previously described patients having one or the other of these rearrangements, including global developmental delay, short stature, hypotonia, delayed puberty, certain facial features and a generalized demyelinating sensory-motor polyneuropathy without clinical manifestation. Our patient also presents some features that were not previously described in relevant individuals, including camptodactyly, preauricular pits and hypertrichosis of the back and elbows.

**Conclusions:**

To our knowledge, this is the first patient to be reported with independent de novo deletion/duplication events involving chromosomes 17 and Y. We discuss possible responsible mechanisms and address the phenotype, particularly in light of the clinical features that were not previously reported for patients bearing a duplication of 17p11.2p12 or a deletion of Yq11. We suggest that some of the previously reported patients with Yq11 deletion and clinical manifestations other than male infertility may have additional chromosomal imbalances that could be identified by chromosome microarray analysis, as illustrated by the present case.

**Electronic supplementary material:**

The online version of this article (10.1186/s13039-019-0438-0) contains supplementary material, which is available to authorized users.

## Background

The 17p11.2p12 locus is an unstable region characterized by the presence of low-copy repeats (LCRs) that predispose the region to acquire several genomic disorders generated by non-allelic homologous recombination (NAHR) [1]. These recurrent genomic rearrangements include: the deletions that cause Smith-Magenis Syndrome (SMS, 17p11.2, ~ 3.6 Mb, MIM **#182290**); the duplications related to Charcot-Marie-Tooth Syndrome type 1A (CMT1A, ~ 1.4 Mb, MIM **#118220**), which is caused by triple gene-dosage for the *PMP22* gene (17p12, MIM *601097) [1–3]; and Potocki–Lupski syndrome (PTLS, ~ 3.6 Mb, MIM **#610883**). The latter is characterized by hypotonia, failure to thrive, reduced body weight, intellectual disability and autistic features; it is believed to be associated with the dosage-sensitive gene, *RAI1* (17p11.2, MIM *607642), which is the only gene known to fall within the 125-kb critical region of this disorder [1, 4]. Most cases of sporadic CMT1A and PTLS (~ 70–80%) are due to NAHR, and the rest can be explained by the DNA replication-based mechanism of fork stalling and template switching (FoSTeS) or the mechanism of microhomology-mediated break-induced replication (MMBIR), which has been implicated in non-recurrent rearrangements that yield widely varied phenotypes [1, 5].

The genes located on the Y chromosome are mainly related to male gonadal determination and spermatogenesis. The euchromatic long arm includes the azoospermia factor (AZF) region. It has been estimated that structural abnormalities of the Y chromosome affect nearly of 1% of the newborn male population [6]; most involve duplication or deletion of Yq heterochromatin [7], and some could be considered normal variants [8].

To our knowledge, only two patients with concurrent de novo deletion/duplication events involving two chromosomes have been reported in the literature [9, 10]; neither case involved both chromosomes 17 and Y, and no pathophysiological mechanism was proposed in either report. Here, we present the first case report of a patient bearing two non-recurrent de novo rearrangements involving a 10.8-Mb duplication of 17p11.2p12 and a 14.7-Mb deletion of Yq11. We show that each arose from the germline of a different parent, discuss possible responsible mechanisms, and address the phenotype, which includes certain clinical features that were not previously described for patients with either mutation alone. Moreover, we suggest that some of the previously reported patients with Yq11 deletion and clinical manifestations other than male infertility may have additional chromosomal imbalances that could be identified by chromosomal microarray analysis (CMA), as illustrated by the present case.

## Case presentation

**Fig. 1:**
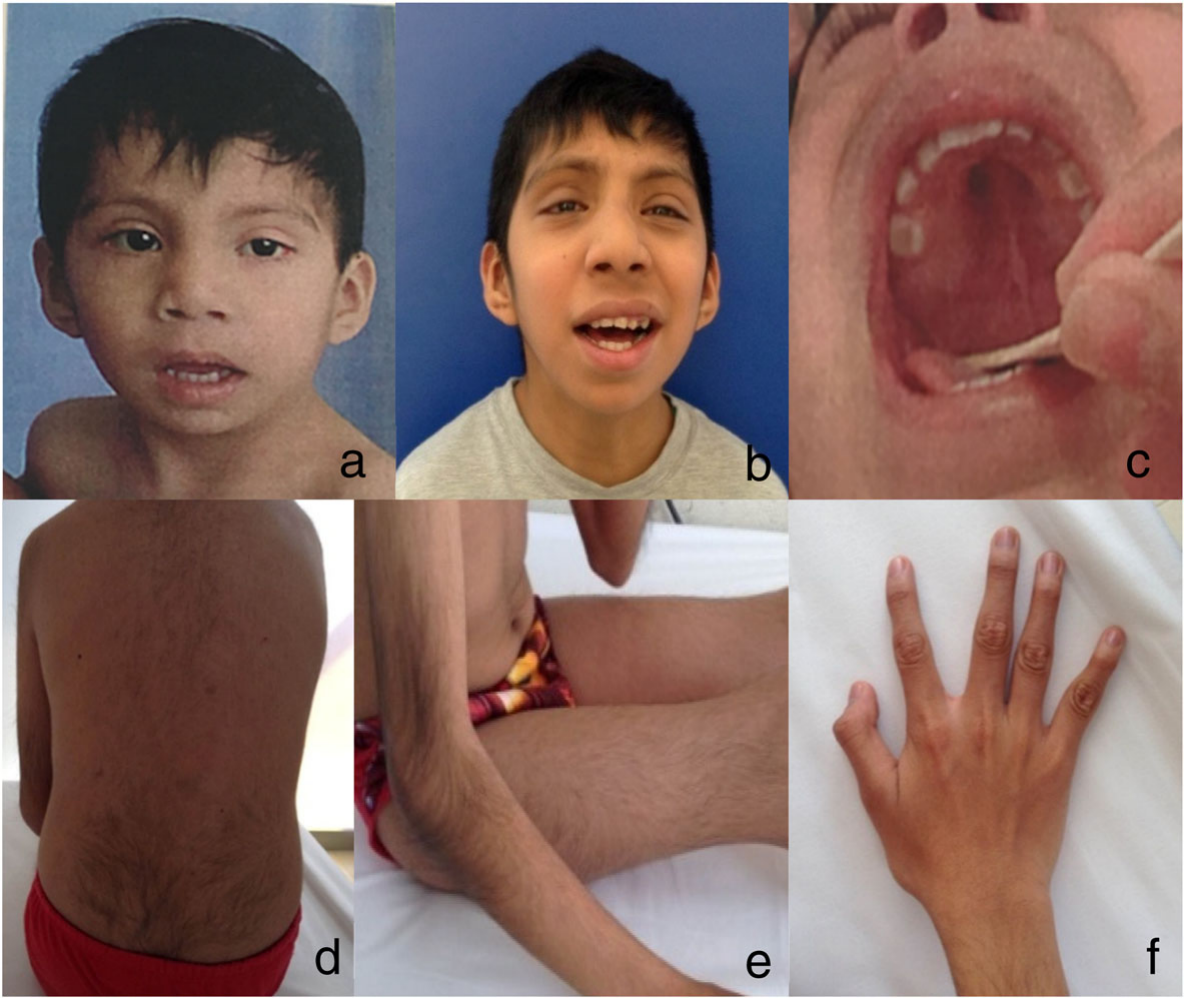
Clinical phenotype of the patient. **a** At 24 months old, showing arched eyebrows, down-slanting palpebral fissures and a large and smooth philtrum. **b** At 15 years old, showing bifrontal narrowing, broad nose, thick and everted lips, a wide chin and large ears. **c** High, arched palate. **d** and **e**) Hypertrichosis of back and elbows. **f** Camptodactyly

We describe a 15-year old boy who is the second child of healthy and non-consanguineous parents. At the time of pregnancy, his mother was 29 years old and his father was 36 years old. He has two healthy brothers and his family and gestational history are unremarkable. Perinatal attention was performed at 41 weeks of gestation after spontaneous vaginal delivery (birth weight, 3.200 kg; length, 51 cm; occipital-frontal circumference and APGAR, unknown). The parents did not remark upon any complication at birth. He was referred to our institute at 24 months of age due to the presence of developmental delay. Physical examination revealed that his weight was 10.200 kg (Z-3.09) and his height was 80 cm (Z-1.16). His head circumference was 46 cm (Z-2.28), and he exhibited bifrontal narrowing, arched eyebrows, down-slanting palpebral fissures (Fig. 1a), bilateral retroauricular pits, global muscular hypotonia and normal external male genitalia. Our clinical approach for assessing global neurodevelopmental delay included brain computed tomography and a basic metabolic screening in dried blood sample, both of which were normal at 2 years of age. At 6 years of age, renal ultrasound and column X-ray were requested because of the presence of retroauricular pits, but the findings were within normal limits. Currently, the patient is 15 years old and has a weight of 31.4 kg (Z-4.17), a height of 1.49 m (Z-2.4) and a head circumference of 51.3 cm (Z-2.9). He has a large and smooth philtrum, thick and everted lips, a wide chin, large ears (Fig. 1b), a high and arched palate (Fig. 1c), hypertrichosis of the elbows and back (Fig. 1d and e) and camptodactyly (Fig. 1f). His pubic development corresponds to Tanner stage II, with delayed genital somatometry (penile length 7 cm, penile volume index 27.75 [Z − 1.86], right testicle 6.91 [Z-3.33], left testicle 6.09 [Z-3.71]). He also exhibits hypotrophy of the extremities, claw toes and decreased distal strength. A bone age assay showed a 2-year delay and hormonal profiling revealed that the values of FSH (0.73 [Ref. 1.0–11 mUI/mL]) and testosterone (85.2 [Ref. 100–1000 ng/dL]) were below the reference ranges, while his LH level (0.85 [Ref. 0.4–7 mUI/mL]) was within the normal range. He has a normal echocardiogram. He achieves self-care, knows numbers from 1 to 20, writes his name and makes sentences of two to three words. In clinical terms, he has a moderate intellectual disability. He is currently homeschooled.

## Results

**Fig. 2:**
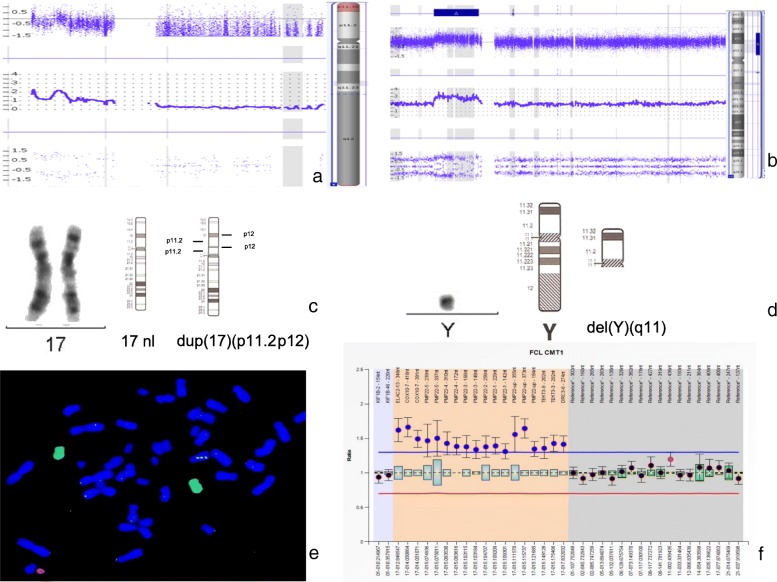
Results of our cytogenetic and molecular studies. **a** CMA plot showing the 14.7-Mb deletion of Yq11 (ChrY: 14,064,952-28,766,705, GRCh37). **b** CMA plot showing the 10.8-Mb duplication of 17p12p11.2 (Chr17:10,701,287-21,504,890, GRCh37). **c** Partial G-band karyotype obtained from peripheral lymphocytes, with 600 GTG bands showing dup(17)(p11.2p12) and **d**) del(Y)(q11). **e** Complete painting of chromosome 17 (green fluorochrome) and the Y chromosome (orange fluorochrome) were performed using Vysis probes (Vysis, USA). **f** MLPA analysis of the *PMP22* gene (SALSA® MLPA® P033-B4 CMT1 probemix; MRC Holland, Amsterdam, The Netherlands), where our patient shows complete duplication of the *PMP22* gene plus the nearby *ELAC2, COX10, TEKT3* and *DRC3* genes

G-band karyotyping of peripheral lymphocytes obtained when the patient was 24 months old revealed 46,X,del(Y)(q11) [20] at a resolution of 450 GTG bands. Multiplex PCR revealed the absence of 21 non-polymorphic sequence-tagged site (STS) markers located in the AZFa, AZFb and AZFc regions, indicating the presence of a terminal Yq deletion (Additional file 1: Figure S1). To delineate the precise deleted genomic Yq interval and to attempt to a phenotype-genotype correlation for our patient, who exhibited unusual clinical manifestations, we performed CMA (Affymetrix CytoScan™ HD) when the patient was 14 years old. This analysis corroborated a 14.7-Mb deletion of Yq11 (ChrY:14,064,952-28,766,705, GRCh37), which included the entire AZFabc region (Fig. 2a). However, unexpectedly, we also identified a 10.8-Mb duplication of 17p11.2p12 (Chr17:10,701,287-21,504,890, GRCh37), which includes the *RAI1* and *PMP22* genes (Fig. 2b). A posteriori lymphocyte high-resolution cytogenetic analysis enabled us to visualize the duplication, yielding 46,X,del(Y)(q11),dup(17)(p11.2p12) (600 bands) (Fig. 2c and d). Chromosome painting by fluorescence in situ hybridization (FISH) of chromosomes 17 and Y yielded normal hybridization [ish (wcpY+,wcp17+); Vysis, USA], ruling out the possibility that both alterations were involved in a structural rearrangement (Fig. 2e). Triple gene dosage of *PMP22* in the proband was confirmed by Multiplex Ligation-dependent Probe Amplification (MLPA; SALSA® MLPA® P033-B4 CMT1 probemix; MRC Holland, Amsterdam, The Netherlands, Fig. 2f). Cytogenetic evaluations of both parents yielded normal karyotypes of 46,XX [15] and 46,XY [15] at 450 GTG bands (Additional file 1: Figure S2), and normal chromosome painting of chromosome 17 (Additional file 1: Figure S3). MLPA also yielded normal results in both parents (Additional file 1: Figure S4). Paternity testing was performed using 15 short tandem repeat markers (13 belonging to the CODIS system), and the results confirmed the proband’s maternity and paternity (data not shown). Genotyping of the tetranucleotide short tandem repeat (STR) marker, *D17S2226*, which is located inside the CMT1A region downstream *PMP22,* indicated that the 17p12 de novo duplication was of maternal origin (Additional file 1: Figure S5).

Having confirmed duplication of the *PMP22* gene, we used directed neurological examination to search for typical clinical manifestations of CMT1A disease, and observed distal hypotrophy without clinical paresthesia, along with a highly arched foot*.* Nerve conduction velocity (NCV) testing showed that the patient had a generalized demyelinating sensory-motor polyneuropathy (motor conduction studies: left median nerve, 24 M/sec; right median nerve, 21 M/sec; left peroneal nerve, 16 M/sec; right peroneal nerve, 15 M/sec; left tibial nerve, 16 M/sec; right tibial nerve, 21 M/sec; normal range > 38 M/sec). Sensory conduction studies in the left and right median, ulnar and sural nerves revealed non-conductible patterns.

## Discussion and conclusions

To the best of our knowledge, this is the first published description of a patient with de novo non-recurrent large deletion/duplication events involving both chromosomes 17 and Y. The 10.8-Mb duplication of 17p11.2p12 includes the *PMP22* and *RAI1* genes, being responsible for Yuan-Harel-Lupski syndrome (YUHAL, MIM **#616652**). Thirty-one YUHAL patients have been reported to date; of them, 22 have de novo duplications [2, 11–16] and the others were not analyzed in this context due to the lack of available parental samples [1]. Clinically, 17/23 YUHAL patients [1] exhibited various symptoms, including demyelinating neuropathy (6/6, not searched in all patients), mild to profound developmental delay (17/17), language delay (15/17), infantile hypotonia (15/17), feeding difficulties (12/17), failure to thrive (11/17), behavioral difficulties (10/17), congenital heart defect (7/17) and renal abnormality (3/17). Facial anomalies were identified in five patients, and included down-slanting palpebral fissures (2/5) with or without hypertelorism (3/5). Additionally, Mendez-Rosado et al. [15] reported a patient sharing this duplication due to an unbalanced insertion in 5p13.1 (Table 1). Our patient shares some clinical characteristics with the above-described patients, including global developmental delay, short stature, hypotonia and some facial features (i.e., down-slanting palpebral fissures); however, he does not present congenital heart disease, renal abnormality or autistic features (he has no clinical difficulty with behavior, social interaction and communication) that have been characteristically reported in 70–100% of patients with PTLS [16]. He also presents some features not previously reported for the relevant patients, such as hypertrichosis of the back and elbows (Fig. 1d and e), camptodactyly (Fig. 1f) and preauricular pits. Our patient does not present obvious symptoms suggestive of peripheral neuropathy despite having a duplication of *PMP22*. However, his NCV test showed a generalized demyelinating sensory-motor polyneuropathy. This is similar to the findings in two previously reported YUHAL patients [12], in which NCV was tested after a molecularly confirmed *PMP22* duplication in the absence of suggestive sensory-motor polyneuropathy manifestations.

**Table 1:** Cytogenetic and/or molecular studies and clinical features reported in patients with de novo duplications of 17p11.2p12 involving *PMP22-RAI1* genes (YUHAL syndrome)

Reference	Cytogenetic/molecular studies	Delineation of the duplication	Shared clinical manifestations								Electrophysio-logical evaluation	Other clinical manifestations
			M	LW	LH	H	D	R/C	AE	DD		
*Lupski* et al.*, 1992* [13]	Cytogenetic analysis dup(17)(p11.2p12) / FISH and DNA markers confirmed the duplication	NA	–	+	+	–	–	+	–	+	Generalized demyelinating sensory-motor polyneuropathy	Abnormal right hand and thumb. VSD
*Upadhyaya* et al.*, 1993* [11]	Cytogenetic analysis dup(17)(p11.2p12) / Southern blot confirmed the duplication	NA	–	–	+	+	+	–	+	+	Generalized demyelinating sensory-motor polyneuropathy	Ear pits
*Roa* et al.*, 1996* [12]	Cytogenetic analysis dup(17)p11 / FISH confirmed duplication	NA	NR	NR	NR	NR	NR	–	NR	+	Generalized demyelinating sensory-motor polyneuropathy	
	Cytogenetic analysis 46,XY,inv. dup(17)(pter→ p11.2::p11.2 → p13.3::p11.2 → qter) / FISH confirmed duplication	NA	NR	NR	NR	NR	NR	+ (1/2)	NR	+	Normal	Complex CHD
*Fernández-Torre* et al.*, 2001* [14]	Cytogenetic analysis 17p(add) / FISH confirmed duplication in 17p13.3	NA	+	+	–	+	+	–	+	+	Demyelinating neuropathy	Divergent strabismus, microphthalmia, hyperactivity
*Potocki* et al.*, 2007* [16]	Cytogenetic analysis dup(17)(p11.2p12) / FISH confirmed duplication / delimited by aCGH	8.2 Mb	NR	+	–	–	–	+	+	+	Peripheral neuropathy	Autistic features, dilated aortic root and bicommissural aortic valve
*Doco-Fenzy* et al.*, 2008* [2]	Cytogenetic analysis dup(17)(p11.2p12) / FISH confirmed duplication	11.15 Mb	+	+	+	+	–	+	–	+	Demyelinating neuropathy	Everted lower lip, VSD, hyperactivity
*Mendez-Rosado* et al.*, 2017* [15]	Cytogenetic analysis / reverse FISH 46,XY,der(5)(5pter- > 5p13.1::17p12- > 17p11.2 or 17p11.2- > 17p12::5p13.1- > 5pter	NA	–	–	–	+	+	–	+	+	Not done	Frontal cortical atrophy, epilepsy
*This patient*	CMA analysis dup(17)(p11.2p12) or arr[hg19] 17p12p11.2(10,701,287-21,504,890)×3 mat. Concurrent Yq11 deletion previously identified by conventional cytogenetics. A posteriori G-banding and MLPA confirmed the 17p duplication.	10.8 Mb	+	+	+	–	+	–	+	+	Generalized demyelinating sensory-motor polyneuropathy	Everted lower lip, retroauricular pits, hypertrichosis

Abbreviations: *aCGH* array comparative genomic hybridization, *AE* Abnormal ears, *CHD* Congenital heart disease, *CMA* Chromosomal microarray analysis, *D* Down-slanted palpebral fissures, *DD* Developmental delay, *FISH* Fluorescence in situ hybridization, *H* Hypertelorism, *LH* Low height, *LW* Low weight, *M* Microcephaly, *NA* Not available, *NR* Not reported, *R/C* Renal/cardiac anomalies, *VSD* Ventricular septal defect

Yuan et al. [1] described that non-recurrent duplications involving *PMP22* and *RAI1* arise because these genes have highly identical LCRs and are located in relatively close proximity to one another (2.5 Mb), and could thus theoretically be involved in a single mutational event that might rely on a mechanism such as FoSTeS/MMBIR. Yuan et al. [1] determined the parental origin of the rearrangements in 14/23 subjects, all of which were found to be de novo [1]. In our patient, *D17S2226* STR marker analysis revealed that the 17p12 duplication was maternal in origin, and the absence of a trialellic pattern suggested the occurrence of an intrachromosomal homologous recombination characteristic of a non-recurrent rearrangement, rather than interchromosomal homologous recombination, which is the main mechanism leading to CMT1A [17].

The 14.7-Mb Yq11 deletion identified in our patient includes the AZF regions necessary to promote and maintain spermatogenesis. The literature includes 23 patients with reported Yq deletions involving the euchromatic region; these patients have other clinical anomalies beyond infertility with a variable clinical spectrum and no clear phenotype-genotype correlation [6–8, 18–29]. The most frequent clinical features are development delay or intellectual disability (15/23), short stature (14/23) and azoospermia (9/23). Other reported characteristics are, in order of frequency: facial dysmorphism (brachycephaly, deep-set eyes, down-slanting palpebral fissures, ptosis, epicanthal folds, high or low nasal bridge, small mouth, high arched or cleft palate, dysmorphic ears; 15/23), cardiac defects (2/23) and hypotonia (2/23) (Table 2). Our patient shows delayed puberty and other manifestations (short stature, developmental delay, facial dysmorphisms and hypotonia); these features have been previously documented in different Yq deletions patients, but no previously reported patient has shown all of the manifestations seen in the present case. Our patient has not undergone any fertility evaluation, but the extent of his Yq deletion suggests that he would be unable to reproduce [30]. As in our patient, most of the previously reported Yq deletions involving the euchromatic region arose as *de novo* events. A number of mechanisms may account for Yq deletion, including Xp;Yq or Y; autosome translocations [18], isochromosome of the short arm (Yp) [19], Xq-Yq interchange in the paternal germline [20] and non-homologous intrachromosomal recombination [21]. However, none of the above-listed mechanisms is thought to explain most Y chromosome deletions, which are non-recurrent, varied in size and do not share a common breakpoint. Few of the previous Yq deletion cases were subjected to a detailed molecular breakpoint analysis, nor were they assessed for other chromosomal aberrations, such as the one found in our patient. Given that infertility is the most frequent (and often the only) manifestation identified in patients with deletions of the Y chromosome, we suggest that some of the previously reported Y-deleted patients with clinical manifestations beyond male infertility (e.g., intellectual disability, short stature and/or dysmorphological features) may have had other chromosomal imbalances. We propose that such imbalances should be searched for with CMA, as they may be missed by conventional cytogenetic analysis, as seen in the present case.

**Table 2:** Cytogenetic and/or molecular studies and clinical features reported in patients with Yq deletions

Reference	Karyotype G, C or Q- banding	Molecular /cytogenomic studies	Extension of the deletion	Shared clinical manifestations									Other clinical manifestations
				M	HP	H	LH	Hy	FD	NMD	A	DD	
*Nakagome* et al.*, 1965* [22]	+	–	NR	**–**	**–**	**–**	+	+	+	+	NA	+	
*Meisner* et al.*, 1972* [19]	+	–	NR	–	–	–	–	–	+	Cryptorchidism	NR	+	Tall stature
*Neu* et al.*, 1973* [23]	+	–	NR	–	–	–	–	–	–	+	+	–	
*Telfer* et al.*, 1973* [24]	+	–	NR	–	–	–	+	–	–	+	NA	+	
*Yunis* et al.*, 1977* [8]	+	–	NR	–	–	–	+	–	–	+	+	–	
*Podruch* et al.*, 1982* [25]	+	–	46,X,del(Y)(q11)	+	–	–	+	–	+	Micropenis	NA	+	
*Kosztolániy* et al.*, 1983* [26]	+	–	46,X,del(Y)(q11)	–	–	–	+	–	–	–	+	+	Gynecomastia
*Langmaid* et al.*, 1974* [27]	+	–	NR	–	–	–	+	–	–	+	NA	–	
*Skare* et al.*, 1990* [21]	+	SB	46,X,del(Y)(q11)	–	+	+	+	–	+	Micropenis	+	–	
*Calzolari* et al.*, 1993* [7]	+	FISH, SB, STS markers	46,X,del (Y)(q11.21qter)	–	–	–	+	+	–	Cryptorchidism	NA	–	CoAo
*Lahn* et al.*, 1994* [20]	+	FISH, STS markers	NR, heterogeneous breakpoints along Yq	+	+	–	–	–	+	Cryptorchidism	+	+	
*Salo* et al.*, 1995* [18] *(9 patients)*	+	STS markers	NR, all patients including Yq11 region	–	5/9	–	5/9	–	8/9	Cryptorchidism 1/9, micropenis 2/9, ASD 1/9	+	9/9	CoAo (*n* = 1)
*Rousseaux-Prévost* et al.*, 1996* [28]	+	STS markers	46,X, del(Y)(q11.21)	–	–	–	–	–	–	+	+	–	
*De Rosa* et al.*, 1997* [29]	+	SB, STS markers	46,X,del(Y)(q11.1pter)	–	–	–	+	–	+	+	+	–	
*Kim* et al.*, 2012* [6]	+	FISH, STS markers	46, X,del (Y)(q11.23)	NR	NR	–	NR	NR	NR	NR	+	NR	
*This patient*	+	STS markers, CMA: arr [hg19] Yq11.21q11.23(14,064,952-28,766,705)×0 pat	46,X,del(Y)(q11)	–	+	+	+	–	+	Micropenis	NA	+	Everted lower lips, Retroauricular pits

Abbreviations: *A* Azoospermia, *ASD* Alteration in sexual differentiation, *CMA* Chromosomal microarray analysis, *CoAo* Coarctation of aorta, *DD* Developmental delay, *FD* Facial dysmorphism, *FISH* Fluorescence in situ hybridization, *H* Hypertrichosis, *Hy* Hypotonia, *HP* High palate, *LH* Low height, *M* Microcephaly, *NA* Not available, *NMD* Normal male development, *NR* Not reported, *SB*: Southern blot analysis, *STS* Sequence-tagged site

There have been a few reported cases involving the simultaneous deletion/duplication of two autosomes due to the presence of a balanced translocation in one parent [31–33]. To our knowledge, there are only two previously published cases of two independent de novo chromosomal rearrangements, such described herein. The first was a child with intellectual disability associated with facial dysmorphism, who exhibited two interstitial rearrangements (16q deletion and 17p duplication) identified by array comparative genomic hybridization (aCGH). Microsatellite analysis of his parents revealed that the non-recurrent 16q deletion (6.29 Mb) was of paternal origin and the non-recurrent 17p duplication (5.89 Mb, not including the *PMP22* gene) was of maternal origin. The authors concluded that the two independent events originated during maternal and paternal meiosis, and seem to have coincided in the patient by chance [9]. The second case was a 6-year-old boy with speech delay, microcephaly and dysmorphic features. He presented a de novo dup (7)(q36.1q36.3) (9.9 Mb) and a del(9)(p24.3) (1.9 Mb) identified by aCGH. His parents presented normal karyotypes and aCGH results, but no further effort was made to identify the parental origin of each alteration. Both 7q duplication and 9p deletion syndromes have been described, but they are heterogeneous and variable in the sizes of the alterations and their clinical manifestations [10].

In summary, this is the first time a 17p12 duplication and Yq deletion have been described in the same individual. The results of our parental karyotype, FISH, MLPA and *D17S2226* analyses suggest that these alterations occurred as independent and de novo events in the proband. However, as we analyzed only leukocytes, we cannot rule out a postzygotic event. Based on our present findings, we suggest that the use of CMA would be justified in patients whose clinical phenotype does not correlate completely with a specific chromosomal rearrangement documented by conventional cytogenetics, as we observed in our patient. This approach could enable researchers to more precisely identify the prevalence of patients with developmental delays and/or dysmorphological phenotypes that should be attributed to two or more independent chromosomal rearrangements.

## Additional file

Additional file 1: Molecular studies performed in the patient and his parents. (DOCX 242 kb)

## Data Availability

All data generated or analyzed during this study are included in the published article and its supplementary information files.
