# Supplementary material for: Report of a patient with a de novo non-recurrent duplication of 17p11.2p12 and Yq11 deletion
Source: Mol Cytogenet. 2019 Aug 1;12:35. doi: 10.1186/s13039-019-0438-0 (PMC6670163; doi:10.1186/s13039-019-0438-0)
Supplement: Supplementary file 1 — Molecular studies performed in the patient and his parents. (DOCX 242 kb) [file 13039_2019_438_MOESM1_ESM.docx]

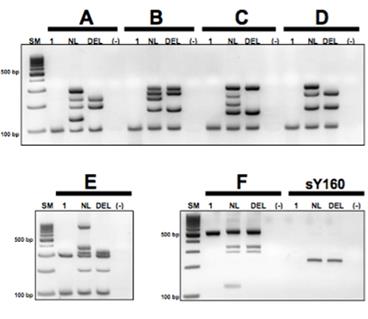

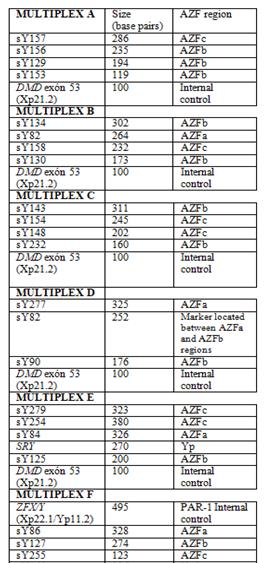


**SUPPLEMENTARY FIGURE 1**. Multiplex PCR reaction for twenty one sY-loci belonging to AZFa, AZFb and AZFc regions; each multiplex reactions including control markers for ZFX/Y, SRY and DMD genes. The location and molecular weight of each amplicon is indicated in the attached table. The sY160 hetrerocromatin marker (Yq12) was amplified separately (236 bp). The analyzed STS included the six non-polymorphic markers from AZFa (sY84, sY86), AZFb (sY127, sY134) and AZFc (sY254, sY255) recommended as STS basic set for screening of Y-chromosomal microdeletions in a clinical setting (Krausz C (2014), et al. Andrology;2(1):5-19). Employed PCR conditions and primers for multiplexing are available upon request. Lane 1: the present patient with deletion of Yq; lane NL: normal control male; lane DEL: control male with a known AZFbc deletion; lane (-): negative control. SM: Size marker, 100 base pair DNA Ladder. Note that the patient does not show any marker for the three AZF regions; instead control fragments of *ZFX/Y*, *SRY* and *DMD* gene-derived amplicons were present. These results along with the absence of the heterochromatin marker sY160, are accordingly with a AZFabc terminal deletion in our patient.


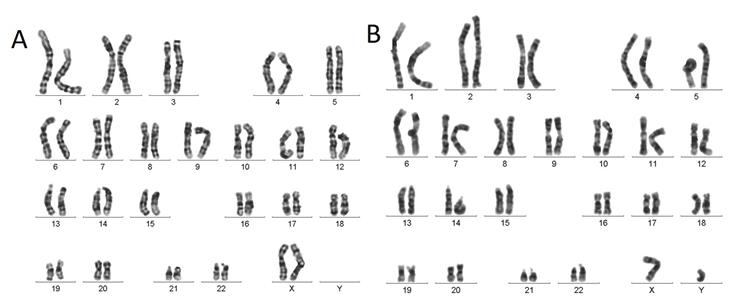


**SUPPLEMENTARY FIGURE 2.** G-band karyotype of peripheral lymphocytes from the patient’s mother (A) and father (B), showing no numerical or structural anomalies, at 450 GTG bands.


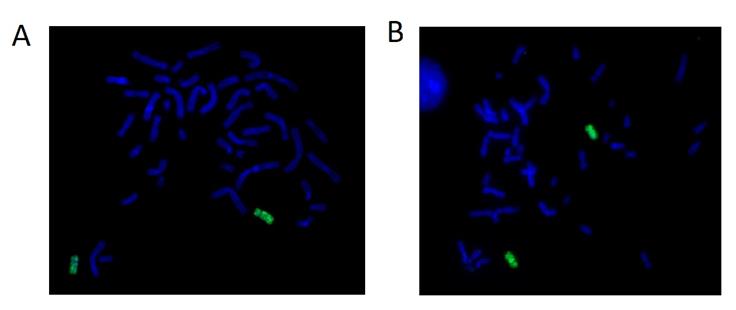


**SUPPLEMENTARY FIGURE 3**. Chromosome painting of chromosome 17 in patient’s mother (A) and father (B), dismissing any balanced structural rearrangement.


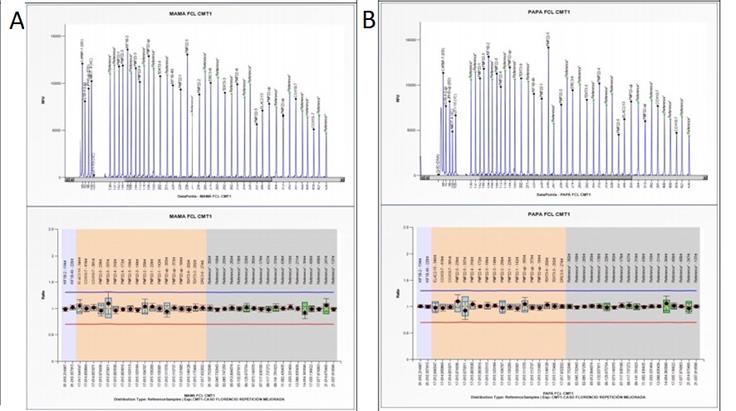


**SUPPLEMENTARY FIGURE 4.** MLPA for Charcot Marie Tooth 1A region, (SALSA® MLPA® probemixes PO33-B3 CMT1, MRC-Holland, MLPA™ ) in patient’s mother (A) and father (B), showing normal doses for *PMP22* gene and other 17p12 region-related loci.

**SUPPLEMENTARY FIGURE 5.** Informative genotypes for the tetranucleotide STR marker, *D17S2226*, in A) the index case (6/12 repeats) and B) his mother (5/12 repeats) and C) his father (6/6 repeats). The index case exhibits an increase in the peak corresponding to the maternally derived 12-repeat allele (estimated dosage ratio: 1.7:1.0), indicating that the 17p12 de novo duplication is of maternal origin.

REFERENCE.

Krausz C et al., 2014. “EAA/EMQN best practice guidelines for molecular diagnosis of Y chromosomal microdeletions: state-of-the-art.” Andrology ;2(1):5-19.
